# When Dark Urine Is Not Nephritis: A Life-Threatening Case of Lipin-1 Deficiency in Infancy

**DOI:** 10.7759/cureus.107990

**Published:** 2026-04-29

**Authors:** Alec Letten, Harry Wong, Nitin Patlolla, Muhammad Junaid Akbar, Haji Sheeraz Khan

**Affiliations:** 1 Paediatrics, Hull University Teaching Hospitals, Hull, GBR; 2 Paediatrics, Leeds Teaching Hospitals NHS Trust, Leeds, GBR; 3 Paediatrics, Sheffield Children's NHS Foundation Trust, Sheffield, GBR; 4 Paediatrics, Hull York Medical School, Hull, GBR

**Keywords:** developmental delay, inherited metabolic disorder, lipin-1 deficiency, metabolism, myoglobinuria, paediatrics, rhabdomyolysis

## Abstract

Lipin-1 deficiency is a rare inherited metabolic disorder and a major genetic cause of severe, recurrent rhabdomyolysis in early childhood, carrying a significant risk of acute kidney injury and mortality if unrecognized. We report the case of a male toddler who first presented at 19 months of age with dark urine and intercurrent viral illness. Initial investigations demonstrated hematuria/myoglobinuria, proteinuria, and markedly elevated transaminases, leading to a presumptive diagnosis of glomerulonephritis. Creatine kinase (CK) was not measured at that time, and the episode was retrospectively felt to represent missed rhabdomyolysis.

The patient subsequently presented similarly with abdominal pain and dark urine when profound rhabdomyolysis was identified by checking CK (CK >120,000 U/L). Aggressive hyperhydration led to biochemical improvement, and genetic testing confirmed a homozygous pathogenic *LPIN1* exon 18 deletion. Since diagnosis, he has experienced multiple recurrent episodes of rhabdomyolysis, predominantly triggered by viral infections, requiring repeated admissions and intensive metabolic management.

Notably, this patient also has a background of global developmental delay (GDD). To our knowledge, this represents the first reported case describing an association between *LPIN1* deficiency and developmental delay. Emerging experimental and clinical evidence suggests a potential biological link between *LPIN1* dysfunction, impaired myelination, and abnormal neurodevelopment, raising the possibility that developmental delay may be an under-recognized phenotypic feature of this condition.

This case highlights the importance of considering rhabdomyolysis in children presenting with dark urine and intercurrent illness and the critical role of early CK measurement in preventing missed diagnoses. Prompt recognition of *LPIN1* deficiency is essential, as delayed treatment may result in life-threatening complications and avoidable morbidity.

## Introduction

Rhabdomyolysis can be a serious and life‑threatening condition that, if left untreated, can result in a cascade of acute kidney injury (AKI), electrolyte disturbances, and disseminated intravascular coagulopathy [1]. Despite the prevalence of rhabdomyolysis, there is no formal definition. A systematic review by Stahl et al. [2] defines rhabdomyolysis as a clinical syndrome of acute muscle weakness, myalgia, and muscle swelling combined with a creatinine kinase (CK) cut-off value of >1000 U/L or >5x the upper limit of normal. In addition, they suggest myoglobinuria and AKI indicate severe rhabdomyolysis.

A single-center, 10-year retrospective case series by Harmer et al. found that infection and trauma are the most common causes of rhabdomyolysis in children, representing 34% and 21% of cases [1]. Inherited metabolic conditions such as lipin-1 deficiency account for 7% of all cases but rise to 12% among cases with CK > 5,000 U/L.

Treatments aim to help prevent or mitigate the progression of AKI [3]. Early and aggressive volume resuscitation forms the foundation of treatment; however, optimal fluid composition and volume remain unclear [4]. Typical regimens use 1.5 to 2.0 times the standard maintenance rate [3]. Other treatment options include urine alkalinisation, diuretics, and renal replacement therapy, but in practice these are uncommon [1,3].

In this report, we describe a case of profound rhabdomyolysis in a toddler caused by lipin-1 deficiency. It is important to identify these patients, as they often present with recurrent severe episodes of rhabdomyolysis, which carry a significant risk of mortality (up to 35%) [5].

## Case presentation

A male toddler was brought to the Paediatric Assessment Unit with coryzal symptoms, lethargy, reduced oral intake and dark urine. He had a past medical history of global developmental delay (GDD), reflux, and cow’s milk protein intolerance. His mother was G1P0 during pregnancy, and the only antenatal concern was maternal gestational diabetes. He was born at 39 weeks and two days, and there were no issues postpartum. There was no family medical history of rhabdomyolysis or metabolic conditions, and no consanguinity.

On first presentation, his urine dip showed haematuria (+++), proteinuria (++) and ketonuria (+++), with nitrites and leucocytes negative. He had a raised alanine aminotransferase (ALT) of 1119 U/L, and the differential diagnoses were glomerulonephritis and sepsis with organ dysfunction. He was started on IV ceftriaxone and had an ultrasound of the urinary tract, which showed normal kidneys and no hydronephrosis, but some thickening of the urinary bladder wall in some views. During his admission, he was noted to have periorbital oedema, and his blood test was positive for Epstein-Barr virus IgM. After three days as an inpatient, his urine colour improved, and ALT decreased, so he was discharged home on seven days of oral antibiotics.

The patient's next presentation was to the local emergency department with a history of acute-onset abdominal pain and dark urine. On examination, he was systemically well, active and alert, but with a congested throat, mild pedal oedema and a non-tender abdomen. Urinalysis showed proteinuria (+++), haematuria (+++) and ketonuria (+) and was negative for leukocytes and nitrites. Respiratory viral polymerase chain reaction was positive for rhino/enterovirus. Subsequently, he was admitted to the paediatric ward, where blood tests showed deranged liver function with an ALT of 871 U/L, and further testing found a significantly raised CK (121,500 U/L), ALT (1385 U/L), lactate dehydrogenase (LDH) (9475 U/L) and urine protein:creatinine ratio (521). Table 1 shows these results. 

**Table 1:** Laboratory results during the first admission and initial period of the second admission ALT: Alanine aminotransferase, CK: Creatine kinase, LDH: Lactate dehydrogenase

Parameter	First admission	Second admission (initial blood tests)	Second admission (further blood tests)	Reference range
ALT (U/L)	1119	871	1385	0-34
CK (U/L)	-	-	121,500	40-320
LDH (U/L)	-	-	9475	215-485
Urine protein:creatinine ratio mg/mmol	-	-	521	<20

Due to the patient's significantly raised CK, he was discussed with the tertiary paediatric metabolic team. On their advice, he was started on a hyperhydration regimen of 1.5 times maintenance fluids, placed on continuous cardiac monitoring, and had twice-daily renal function and an urgent genetic test sent. This treatment regimen was successful with symptomatic improvement and CK levels improving over five days.

Once the CK dropped below 9000, the IV fluids were stopped. Unfortunately, within 48 hours, there was a significant rebound of CK levels to 26,300 U/L, which necessitated IV fluids being restarted. Due to difficulty with IV access, it was decided that central access would be beneficial to optimise management; however, due to the developmental delay and his age, this required sedation. There were concerns from the metabolic team that he was at high risk of malignant hyperthermia, but, after discussions between the metabolic team and paediatric anaesthetists, the patient underwent a general anaesthetic for insertion of a central venous line. This was done using ketamine, atracurium, and midazolam.

**Figure 1:**
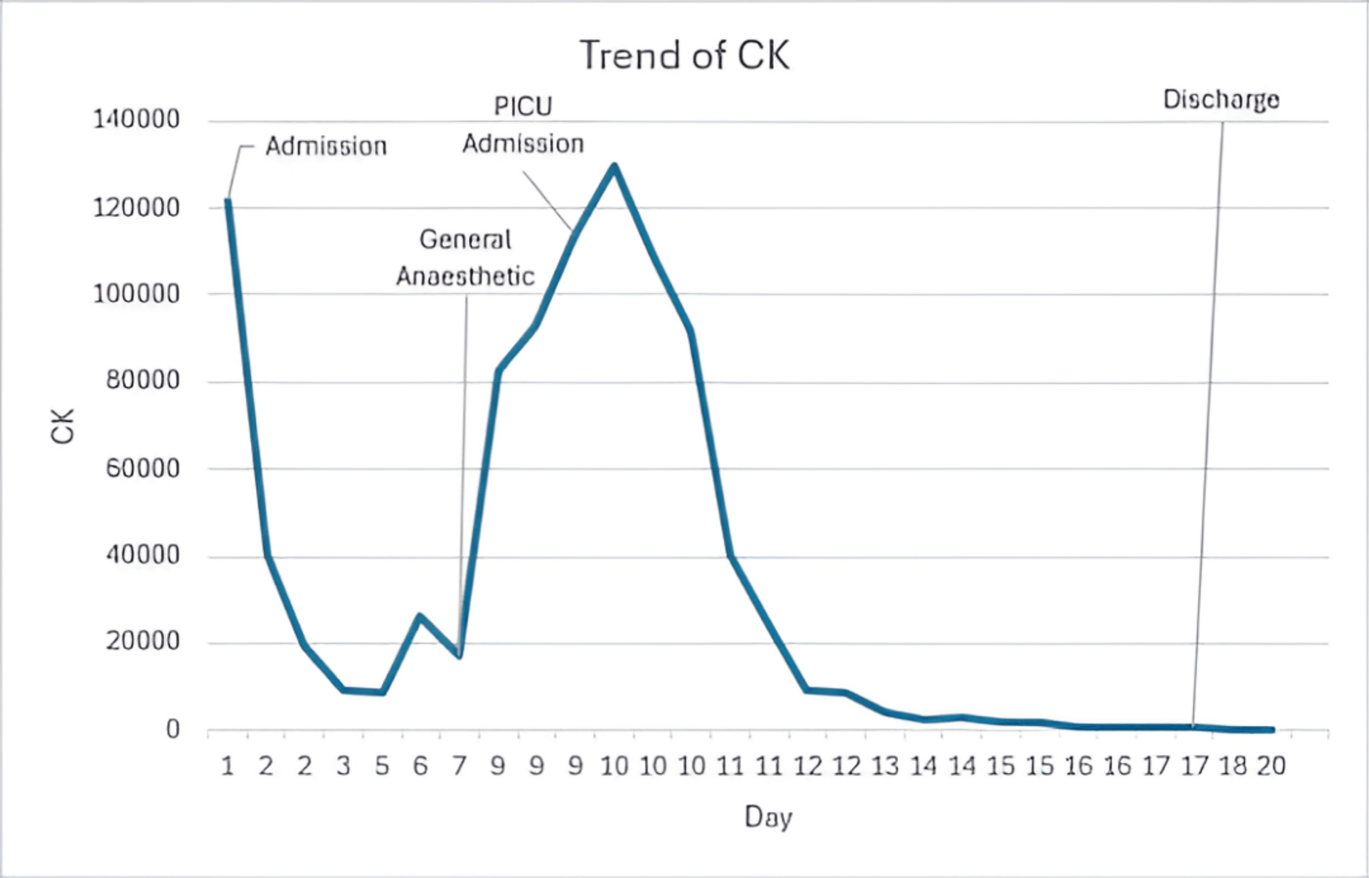
Trend of patient's CK on first admission CK: Creatine kinase

The procedure was successful but complicated by a rising CK postoperatively despite 1.5 times hyperhydration therapy and postintubation stridor requiring a dose of dexamethasone. Due to the uncontrolled rise in CK, the patient was transferred to the paediatric ICU for a three-day course of methylprednisolone. Following this treatment and a two-time maintenance IV fluid regimen, the CK dropped below 4,000 U/L, and the patient was able to be discharged home. Follow-up tests showed CK normalised and ALT significantly improved within 12 days of discharge. Figure 1 shows the trend of CK throughout the patient's admission.

Genetic testing used a trio gene-agnostic genome sequence analysis (known in the UK as an R14 panel) [6]. This demonstrated a homozygous pathogenic deletion of exon 18 in the *LPIN1 *gene. This has been described as a cause of recurrent severe rhabdomyolysis in infancy [7]. As this was a trio sequence, it also identified that his mother and father are unaffected carriers of this LPIN1 gene deletion.

Since the patient's initial diagnosis of* LPIN1* deficiency in late 2023, he has had a further eight admissions with rhabdomyolysis. In multiple instances, the trigger was rhino/enterovirus, with an initial presentation of coryzal symptoms, reduced oral intake and sometimes fever. Less frequently, he presented with stiffness of the lower legs and a reluctance to bear weight. A singular episode of rhabdomyolysis was caused by a general anaesthetic, which was required for the insertion of a totally implantable venous access device and percutaneous endoscopic gastrostomy.

Management during admissions is determined in conjunction with the tertiary hospital metabolic team. During the initial stages of becoming unwell, the patient is given an oral high-carbohydrate supplement (Polycal powder) at home. This provides an alternative source of energy to prevent the catabolism of skeletal muscles, thereby reducing the frequency and severity of rhabdomyolysis episodes [8]. 

## Discussion

A literature review was conducted to identify papers relating to rhabdomyolysis, using the keywords rhabdomyolysis and *LPIN1* to narrow the search. The databases searched were Medline and Embase, with no date restriction or limits. Abstracts were reviewed to identify those relating to *LPIN1 *deficiency specifically. To better appreciate the underlying pathophysiology, review articles were prioritised, as these provide the strongest evidence, given that they are the synthesis of knowledge from multiple studies and thus are likely to be more comprehensive and less biased.

Table 2 outlines the possible aetiologies of rhabdomyolysis, with metabolic causes contributing to 7% of all cases [1]. A homozygous *LPIN1 *mutation has been identified as the second most common genetic cause of rhabdomyolysis and, subsequently, severe myoglobinuria in early childhood [7]. Homozygous deletion of exon 18, as seen in our patient, occurs in 86% of Caucasian patients [9]. Homozygous mutations are linked with increased childhood rhabdomyolysis with a median age of 21 months, very similar to this patient, aged 19 months at first presentation.

**Table 2:** Causes of rhabdomyolysis CK: Creatine kinase [1]

Aetiology	All cases	Non-severe (CK <5000)	Severe (CK >5000)
Infective	34%	27%	29%
Trauma	21%	21%	12%
Secondary to seizure	11%	12%	7%
Metabolic	7%	10%	12%
Drug-induced	10%	7%	9%
Immune-mediated	2%	10%	5%
Muscular dystrophies	2%	5%	17%
Other	13%	8%	9%

What is *LPIN1*?

The *LPIN1 *codes for the protein lipin-1, which is an enzyme and transcriptional factor highly expressed in skeletal muscle and adipose tissue. The specific pathophysiology of rhabdomyolysis in patients with LPIN1 deficiency is still unknown in humans, but studies on mice can provide us with a better idea of the possible mechanisms. Lipin-1 acts as phosphatidic acid phosphatase to convert phosphatidic acid into diacylglycerol [10], which is needed for triacylglycerol (TAG) synthesis [11]. The TAGs are required for energy storage between meals in the form of fat, stored in adipocytes, so with a deficiency in lipin-1, the ability to create TAGs is reduced. The impairment in lipid metabolism is exacerbated by a pro-inflammatory response. Studies in biopsies of skeletal muscle, myoblasts and myotubes have identified that proinflammatory cytokines, specifically tumour necrosis factor alpha (TNFα) and interleukin-1 beta (IL-1β), result in a reduction in the expression of the *LPIN1 *gene; however, they also stimulate an increased production of lipid droplets within cells. This may be due to upregulation of lipin-2, but excessive numbers of lipid droplets can trigger autophagy [12].

Furthermore, lipin-1 deficiency has been linked to the accumulation of abnormal and dysfunctional mitochondria due to an impairment of the normal process of mitophagy (destruction of mitochondria by autophagy) [13]. The combination of all these effects, impaired fatty acid metabolism, dysfunctional autophagy and an uncontrolled inflammatory response, contributes to the breakdown of myoblasts. This releases CK and myoglobin into circulation, leading to rhabdomyolysis [14].

Management of *LPIN1* deficiency

The mainstay of management is fluid resuscitation. The rate of IV fluid is determined based on clinical response and the presence of other features such as hyperkalaemia, which would necessitate more aggressive fluid resuscitation [15]. Hyperhydration promotes renal clearance and facilitates the excretion of CK. A further management option is the use of steroids due to their anti-inflammatory nature [16], and in Michot’s study of myoblasts, the addition of dexamethasone countered the pro-inflammatory response and decreased the number of lipid droplets in cells [12]. Serial measurement of CK is also recommended to both monitor the response to treatment and guide the ongoing management [15].

Retrospectively reviewing this patient's first presentation, the dark urine, proteinuria and haematuria prompted investigations for nephritis. Although CK is not part of the recommended diagnostic workup for suspected nephritis [17], this simple investigation could have facilitated earlier diagnosis.

*LPIN1* and GDD

Our patient had a background of GDD. To our knowledge, this is the first case report that describes a patient with *LPIN1 *deficiency and GDD. Novel heterozygous mutations in *LPIN1*, identified in a family with adult-onset myasthenia gravis and peripheral neuropathy, were replicated in zebrafish embryos to assess the effect on the myotome. This demonstrated defects in myelination [18]. A lack of myelination has previously been identified in children with GDD compared to those without [19], with one study quoting a difference equivalent to a 3.2-year delay in myelination for those with GDD [20]. Taken together, these findings indicate that mutations in the* LPIN1* gene may interfere with normal myelination and thus with the normal process of neurodevelopment. This, therefore, suggests the presence of GDD in this patient that may be an under-recognised phenotypic manifestation of the LPIN1 deficiency rather than a coincidental comorbidity. Current evidence for this is limited, and alternative explanations remain possible. Further dedicated research and case reports are needed to clarify the role of LPIN1 in neurodevelopment.

## Conclusions

This case report details a toddler with a background of LPIN1 deficiency who has had recurrent episodes of rhabdomyolysis requiring admission and treatment with hyperhydration. The crucial takeaway from this case is that for a patient presenting with dark-coloured urine and an inconclusive initial examination and investigation, a CK level should be obtained. The first admission of this patient, treated as glomerulonephritis, was most likely the first presentation of rhabdomyolysis; however, with no CK level taken, it was missed. Failure to treat rhabdomyolysis would lead to cumulative kidney injury and a greater risk of mortality. As the kidneys are damaged over sequential episodes, baseline kidney function becomes deranged, making future AKI harder to identify. Furthermore, with the background of developmental delay, key elements of the history, such as pain and stiffness, which would aid a diagnosis, may not be elucidated, making measurement of CK even more imperative.

## References

[1] Harmer MJ, Nijloveanu V, Thodi E (2023). Paediatric rhabdomyolysis: a UK centre's 10-year retrospective experience. J Paediatr Child Health.

[2] Stahl K, Rastelli E, Schoser B (2020). A systematic review on the definition of rhabdomyolysis. J Neurol.

[3] Kuok MC, Chan WK (2025). Rhabdomyolysis in children: a state-of-the-art review. Children (Basel).

[4] Bosch X, Poch E, Grau JM (2009). Rhabdomyolysis and acute kidney injury. N Engl J Med.

[5] Tein I, DiMauro S, DeVivo DC (1990). Recurrent childhood myoglobinuria. Adv Pediatr.

[6] (2026). R14 rapid genome sequencing service Exeter Clinical Laboratory International. https://www.exeterlaboratory.com/genetics/genome-sequencing/.

[7] Michot C, Hubert L, Brivet M (2010). LPIN1 gene mutations: a major cause of severe rhabdomyolysis in early childhood. Hum Mutat.

[8] Indika NL, Vidanapathirana DM, Jasinge E, Waduge R, Shyamali NL, Perera PP (2020). Lipin-1 deficiency-associated recurrent rhabdomyolysis and exercise-induced myalgia persisting into adulthood: a case report and review of literature. Case Rep Med.

[9] Meijer IA, Sasarman F, Maftei C (2015). LPIN1 deficiency with severe recurrent rhabdomyolysis and persistent elevation of creatine kinase levels due to chromosome 2 maternal isodisomy. Mol Genet Metab Rep.

[10] Han GS, Wu WI, Carman GM (2006). The Saccharomyces cerevisiae lipin homolog is a Mg2+-dependent phosphatidate phosphatase enzyme. J Biol Chem.

[11] Csaki LS, Dwyer JR, Fong LG, Tontonoz P, Young SG, Reue K (2013). Lipins, lipinopathies, and the modulation of cellular lipid storage and signaling. Prog Lipid Res.

[12] Michot C, Mamoune A, Vamecq J (2013). Combination of lipid metabolism alterations and their sensitivity to inflammatory cytokines in human lipin-1-deficient myoblasts. Biochim Biophys Acta.

[13] Schweitzer GG, Collier SL, Chen Z (2019). Loss of lipin 1-mediated phosphatidic acid phosphohydrolase activity in muscle leads to skeletal myopathy in mice. FASEB J.

[14] Kahraman AB, Karakaya B, Yıldız Y (2022). Two tales of LPIN1 deficiency: from fatal rhabdomyolysis to favorable outcome of acute compartment syndrome. Neuromuscul Disord.

[15] Rout P, Chippa V, Adigun R (2025). Rhabdomyolysis. StatPearls [Internet].

[16] Tuchmann-Durand C, Roda C, Renard P (2023). Systemic corticosteroids for the treatment of acute episodes of rhabdomyolysis in lipin-1-deficient patients. J Inherit Metab Dis.

[17] Welsh Clinical Network for Paediatric Nephrology. (2020 (2025). Guidelines for the management of a child with haematuria. G. Smith, Ed.) [PDF]. University Hospital of.

[18] Lu S, Lyu Z, Wang Z (2021). Lipin 1 deficiency causes adult-onset myasthenia with motor neuron dysfunction in humans and neuromuscular junction defects in zebrafish. Theranostics.

[19] Ha SY, Sung YH (2021). Changes of neural pathways after Vojta approach in a child with developmental delay. Children (Basel).

[20] Pujol J, López-Sala A, Sebastián-Gallés N (2004). Delayed myelination in children with developmental delay detected by volumetric MRI. Neuroimage.

